# Retraction: Dopaminergic regeneration by neurturin-overexpressing c17.2 neural stem cells in a rat model of Parkinson's disease

**DOI:** 10.1186/1750-1326-4-45

**Published:** 2009-11-04

**Authors:** Wei-Guo Liu, Xi-Jing Wang, Guo-Qiang Lu, Biao Li, Gang Wang, Sheng-Di Chen

**Affiliations:** 1 Department of Neurology & Neuroscience Institute, Ruijin Hospital, Shanghai Jiaotong University School of Medicine, Shanghai 200025, PR China; 2 Lab of Neurodegenerative Diseases, Institute of Health Science, Shanghai Institutes of Biological Sciences, Chinese Academy of Science & Shanghai Jiaotong University School of Medicine, Shanghai 200025, PR China

## Retraction

This article [1] was submitted to *Molecular Neurodegeneration *without the knowledge of all authors. In addition an inappropriate figure was included and so the authors would like to retract the article. Dr Liu would like to extend an apology to the co-authors and readers for any inconvenience caused.
